# Long-Lasting Therapies with High Doses of D-chiro-inositol: The Downside

**DOI:** 10.3390/jcm12010390

**Published:** 2023-01-03

**Authors:** Maurizio Nordio, Maria Salomè Bezerra Espinola, Gabriele Bilotta, Elena Capoccia, Mario Montanino Oliva

**Affiliations:** 1 Department of Experimental Medicine, University “Sapienza”, 00161 Rome, Italy; 2 The Experts Group on Inositol in Basic and Clinical Research (EGOI), 00156 Rome, Italy; 3 Systems Biology Group Lab, 00161 Rome, Italy; 4 Alma Res Fertility Center, 00198 Rome, Italy; 5 Department of Obstetrics and Gynecology, Santo Spirito Hospital, 00193 Rome, Italy

**Keywords:** inositol, D-chiro-inositol, fertility, oligomenorrhea, amenorrhea, testosterone, asprosin, insulin sensitivity

## Abstract

Background: Recent studies reported possible concerns following long-lasting treatments with high doses of D-chiro-inositol in women. However, to date, no clinical trial has investigated or validated these concerns. We addressed this issue both retrospectively and with a prospective pilot study. Methods: For the retrospective analysis, we searched our databases for insulin-resistant women who took 1200 mg/day D-chiro-inositol for 6 months. In our prospective study, we enrolled 10 healthy women to supplement with the same therapeutic scheme. We performed statistical analyses through the Wilcoxon Signed-Rank Test. A *p*-value < 0.05 was considered significant. Results: Twenty women underwent 6 months of 1200 mg/day D-chiro-inositol. The treatment significantly decreased BMI, glycemia, insulinemia, HOMA-IR, serum levels of LH, total testosterone, and DHEAS. Serum estradiol rose and menstrual abnormalities occurred following the treatment. In our prospective study, we observed increases in serum levels of total testosterone and asprosin in healthy women. Conclusions: This is the first clinical evidence demonstrating that long-term treatments with high dosages of D-chiro-inositol can predispose women to hormonal and menstrual abnormalities. Moreover, the accumulation of D-chiro-inositol following such treatment regimen may lead to detrimental effects in non-reproductive tissues, as demonstrated by the increase in asprosin levels.

## 1. Introduction

Inositols are natural compounds occurring both in animals and in plants as pivotal molecules in signaling processes. Nine possible stereoisomers exist, among which myo-inositol (MI) is the most abundant, representing more than 99% of the total inositol content, while D-chiro-inositol (DCI) is the second most represented isomer. Inositols are relevant molecules in both basic and clinical research as they participate in several pathways of primary importance, including pathways of insulin, insulin-like growth factors, and gonadotropins. Indeed, each of these hormones activates inositol signaling to a different extent and with isomer specificity, as, for example, FSH triggers only MI signaling [1].

Since they participate in insulin signaling, inositols are used as food supplements to improve insulin sensitivity in patients with insulin resistance or type 2 diabetes [2]. Insulin resistance is a common feature of Polycystic Ovary Syndrome (PCOS), a condition diagnosed given the presence of two out of the three following criteria: anovulation, hyperandrogenism, and cystic ovaries. Currently, inositols are considered effective and safe compounds to treat PCOS signs and symptoms, as they are involved in several pathways that appear deregulated in this syndrome [3]. Among the dosages proposed over the years, MI treatment is recommended at 4000 mg per day, while literature evidence on DCI reports about 1000 mg per day [4,5].

Recent literature reports have suggested possible detrimental effects for long treatments with high dosages of DCI, due to its molecular activities [3,6,7]. In fact, DCI acts as a transcriptional inhibitor of aromatase, the enzyme that converts androgens into estrogens, thus promoting the accumulation of androgens. As women with PCOS usually experience hyperandrogenism and an excess of DCI in the ovaries, they can suffer the unfavorable effects of prolonged treatments with high doses of DCI, impairing the health of their ovaries and exacerbating their androgen excess. Therefore, several papers suggest prescribing DCI only in specific cases and to avoid long-lasting therapies with high dosages (Table 1) [3,6,8] in order to improve insulin sensitivity and prevent the effects on steroidogenesis. Moreover, a recent study reported that high doses of DCI may exert detrimental effects in a mouse model. Indeed, the ovaries of treated mice displayed histological features resembling those of women with PCOS [9], suggesting that the use of high doses of DCI for prolonged therapies should be carefully evaluated.

Considering the unpleasant effects observed in mice, we decided to investigate whether DCI could also induce a PCOS-like condition in women. Therefore, we retrospectively analyzed data from patients who took high doses of DCI for long-lasting treatment of insulin resistance. Almost all patients reported menstrual abnormalities, and we hypothesized that these are adverse effects due to D-chiro-inositol treatment. In fact, as DCI enhances the insulin signal, the detrimental effects could prevail once insulin sensitivity is restored. We searched the literature to identify an early marker of metabolic abnormalities. We selected asprosin, which is a recently discovered protein involved in various metabolic pathways, including glucose release in plasma from the liver and appetite control in the hypothalamus [10]. Several recent studies reported that patients with PCOS display increased systemic asprosin levels with respect to healthy controls [11,12]. The available evidence suggests that increased plasma levels of asprosin correlate with insulin resistance [11,13]. Since we did not have retrospective data on asprosin available, we designed a prospective study treating healthy insulin-sensitive women with high doses of DCI. Therefore, we included asprosin among the outcomes of our prospective study as a hallmark of metabolic alterations and a putative prognostic factor for the induction of PCOS-like conditions.

## 2. Materials and Methods

### 2.1. Database Analyses

We searched our medical databases for insulin-resistant women who took DCI as an insulin sensitizer. We included 20 patients that met the following criteria: age between 18 and 50 years; mild-to-severe hyperglycemia defined as glycemia greater than or equal to 100 mg/dL; insulin resistance defined as Homeostasis Model Assessment of Insulin Resistance index (HOMA-IR) greater than or equal to 2.5; prescribed treatment with 1200 mg/die DCI. We excluded patients who met any of the following criteria: pregnancy; delivery in the previous 6 months; menopause, either natural or iatrogenic; alcohol or drug abuse; overt diabetes; tumor lesions; treatment with drugs; treatment with other supplements containing inositols; steroidal hormone unbalance.

### 2.2. Retrospective Outcomes

We compared retrospective outcomes collected before starting the supplementation with 1200 mg/die of DCI and after 6 months of treatment. We evaluated the following parameters: Body-mass Index (BMI), glycemia, insulinemia, HOMA-IR index, Follicle-Stimulating Hormone (FSH), Luteinizing Hormone (LH), free Testosterone (T), Estradiol, De-Hydro-Epi-Androsterone Sulfate (DHEAS), and the appearance of menstrual abnormalities (oligomenorrhea or amenorrhea).

### 2.3. Prospective Study

Ten healthy women referred to the Alma Res Fertility Center in Rome (Italy) and to the A.S.L. RMF in Civitavecchia (Italy) for routine analyses between March and July 2022 and were enrolled in July 2022. All women enrolled gave their written informed consent after the explanation of the study purpose. The study was conducted following the Ethical Principles of the Declaration of Helsinki and the national laws and was approved by the Internal Review Board of Clinical Alma Res, Protocol No. 007/2022. The study was registered at http://clinicaltrials.gov (accessed on 7 April 2022), code NCT05448378. The inclusion criteria were the following: age between 18 and 50 years, good state of health, and a regular menstrual cycle. The exclusion criteria were the following: pregnancy; currently breastfeeding; menopause; alcohol or drug abuse; insulin resistance defined as HOMA-IR index greater than or equal to 2.5; other medical morbidities, such as hypertension, PCOS, or diabetes; oligomenorrhea or amenorrhea; current treatment with corticosteroids or hormones; or the use of Gonadotropin-Releasing Hormone (GnRH) analogues, Selective Estrogen Receptor Modulators (SERMs), or Selective Progesterone Receptor Modulators (SPRMs) within the previous 6 months. The enrolled women received one tablet containing 600 mg of DCI twice per day for 6 months. We examined the patients after 1 month of treatment, and we intended to also evaluate their parameters at the end of the 6-month supplementation. Due to the reported effects, the trial was terminated early for ethical reasons, preventing the recording and analyses of 6-month supplementation.

### 2.4. Prospective Outcomes

We considered as the primary outcome the change in free T levels. The secondary outcomes were variations in the following parameters: BMI, glycemia, insulinemia, HOMA-IR, FSH, LH, estradiol, DHEAS, and asprosin. The expected time points for the analyses were the baseline and after 1 and 6 months of treatment. Blood samples were collected on day 4 from the beginning of the cycle.

### 2.5. Statistical Analyses

Statistical analyses comparing variables in the same group at different times were performed using the Wilcoxon Signed-Rank Test. Values are indicated as the median [25th percentile–75th percentile]. A *p*-value<0.05 was considered statistically significant.

## 3. Results

### 3.1. Retrospective Analysis

We found complete data belonging to 20 women who fulfilled the inclusion criteria and did not meet any of the exclusion criteria. The clinical features of these patients at baseline and after 6 months of DCI treatment are reported in Table 2. After the supplementation, 16 women experienced a reduction in BMI, 17 presented a reduction in glycemia, while all women displayed reduced insulinemia and HOMA-IR index. Considering the hormonal profile, 14 women displayed reduced LH, 19 women achieved a reduction in free T, and 18 presented reduced DHEAS. On the contrary, estradiol levels rose in 16 patients. it is noteworthy that, while at baseline only 4 women displayed oligomenorrhea or amenorrhea, 16 women reported menstrual abnormalities (i.e., oligomenorrhea or amenorrhea) after 6 months of treatment.

### 3.2. Prospective Study

On the basis provided by the retrospective evaluation, we decided to test the safety of prolonged treatment with an analogous dose of DCI in healthy subjects. Therefore, we enrolled 10 healthy women, whose clinical features at baseline and after one month of DCI supplementation are summarized in Table 3. All these women, after only one month of supplementation with 1200 mg/die of DCI, displayed a significant increase in free T levels. The same trend was observed for asprosin, which was significantly higher after one month in all the subjects. Bearing in mind the potential concerns underlying these observations, we deemed it appropriate to terminate the treatment early for ethical reasons.

## 4. Discussion

We found that unrequired treatments with DCI can lead to detrimental effects in women. In the latest years, several papers have pointed out that long-lasting supplementation with high doses of DCI can exert detrimental effects, especially in certain kinds of patients [3,6,14]. This is mainly related to the role of DCI as a transcriptional inhibitor of aromatase, which oxidizes androgens [15]. As a consequence, androgens accumulate, inducing local hyperandrogenism and worsening ovarian functionality, such as in patients with PCOS.

The first connection between high doses of DCI and the worsening of PCOS came from two preclinical studies from Bevilacqua and colleagues. In the first one [16], the authors reported that the supplementation with various MI/DCI ratios differently impacted the ovarian physiology in a mouse model of PCOS. Indeed, their findings highlighted that ratios with a higher MI amount led to favorable outcomes, with the 40:1 ratio representing the best to recover a near-physiological condition. On the contrary, the ratios with higher DCI content were less effective in restoring ovarian activity, also showing minor harmful effects. The second study [9] further characterized the effects of high doses of DCI on the ovary. Indeed, they demonstrated for the first time that the ovaries of mice receiving high doses of DCI, corresponding to 1200 mg/day in humans, for five estrous cycles, displayed a PCO-like histology. Moreover, in this case, they found that DCI caused cystic follicles and oocyte deprivation, also halting cycle progression.

Despite these preclinical data suggesting that such adverse effects may also occur in women following long treatments with high dosages of DCI, to date, no clinical study had ever validated these concerns. Herein, we report that 1200 mg of DCI per day for 6 months induced menstrual abnormalities in women. Particularly, in our retrospective study, 80% of patients who took 1200 mg/day of DCI to treat insulin resistance and hyperglycemia reported oligomenorrhea and amenorrhea after a 6-month supplementation. To the best of our knowledge, this is the first clinical evidence pointing out that the effects of DCI depend on the duration and the dose of supplementation. These findings are in line with our previous data collected after treating women with PCOS with different inositol ratios [17]. In that study, an increase in DCI amount likely hampered MI efficacy. 

However, there are also successful reports in the literature regarding DCI supplementations in terms of recovery in metabolic balance and ovarian functionality. A trial from Nestler et al. [4] involving obese insulin-resistant patients with PCOS demonstrated that a 6-week-long treatment with 1200 mg/day of DCI restored ovulation in 19 out of 22 patients (86%) compared to 6 out of 22 patients taking placebo (27%). These results have been attributed to the insulin-sensitizing effects of DCI, which reduce systemic insulin levels, removing its overburdening stimulus on the ovaries. In fact, Larner [18] demonstrated that insulin prompts the conversion of MI to DCI, granting tissue-specific ratios of the two isomers. He also demonstrated that such conversion is impaired in insulin resistance conditions; therefore, insulin-resistant patients display higher MI/DCI ratios in their tissues. Both Unfer [19] and Heimark [20] later observed that ovaries exhibit a different behavior, with an MI/DCI ratio that decreases in the case of insulin resistance and PCOS, resulting in larger quantities of DCI. Their data indicate that ovaries fail to become insulin resistant, remaining sensitive to insulin and, thus, overburdened by compensatory systemic hyperinsulinemia [21]. Given the correlation between insulin resistance and the higher amount of ovarian DCI, it is likely that in our retrospective study, the supplementation further enriched the ovaries with DCI, impairing or halting the menstrual cycle.

If, on the one hand, DCI displays beneficial activities on the insulin pathway, on the other hand, we should acknowledge that long treatments with high DCI dosages may exert negative effects on the ovaries. As such, our data reaffirmed that DCI preferentially recovers insulin sensitivity in case of insulin resistance. Otherwise, once the correct metabolism is restored, or in the case of normo-insulinemic patients, high doses of DCI find no applications. Indeed, in those cases, patients experience DCI’s negative effects on the ovaries. In our retrospective study, patients were prescribed treatment with DCI because of their insulin resistance. However, after 6 months of treatment with high doses of DCI, they reported menstrual abnormalities. We hypothesize that after restoring insulin sensitivity, DCI administration altered the MI/DCI ratio in the ovaries, resulting in local depletion of MI. This condition desensitizes the ovaries to FSH signaling, resulting in menstrual irregularities. As further evidence, our prospective analysis demonstrated that supplementation with high doses of DCI in healthy subjects increases T levels toward the upper limits of physiological values, likely due to DCI accumulation in the ovaries.

The ovaries are the primary source of T in women, while only a minor part derives from the adrenal glands [22]. This means that the effect on T production may derive from the activity of DCI. As proof, previous in vitro evidence already highlighted that treating human ovarian cells with DCI resulted in T overabundance [23], likely due to transcriptional inhibition of aromatase [15]. Moreover, our data on T levels matched the findings by Monastra et al., who observed similar variations in healthy male volunteers [24]. The increase in asprosin production clearly suggests a systemic effect, as this protein is synthesized in the adipose tissue. The available evidence relates higher asprosin plasma levels with impaired insulin sensitivity in the case of gestational diabetes mellitus, type 2 diabetes, and PCOS [11,13]. However, there is still insufficient knowledge in the literature on whether asprosin is an etiological factor of these pathologies or just a consequence of their development.

Molecules that restore a euglycemic state should lower plasma levels of markers for altered metabolism. Unexpectedly, a 30-day treatment with an insulin sensitizer such as DCI increased the concentration of asprosin, which is considered a novel early marker of altered metabolism. The mechanisms underlying this effect are still unclear, but the clinical outcomes seem to suggest that excess DCI can have harmful effects in non-reproductive tissues as well, and this deserves further investigations. The present study does not clarify whether DCI accumulation or lack of MI is responsible for what we observed, but we can confidently point out that the recorded effects correlate with an altered MI/DCI ratio in favor of DCI. Nonetheless, this is a preliminary pilot study, and these data must be validated through larger randomized and controlled studies to confirm their significance and in vitro experiments to assess the molecular mechanisms.

## 5. Conclusions

This study provides the first demonstration that long treatments with high doses of DCI alter the functionality of both reproductive and non-reproductive tissues, affecting menstrual regularity and increasing testosterone and asprosin levels. These results seem to confirm previous hypotheses, namely, that time and dose represent two pivotal elements to consider when prescribing DCI-based therapies. Although the evidence herein needs to be validated in larger randomized and placebo-controlled studies, it certainly plants a seed of doubt that clinicians should no longer ignore.

## Figures and Tables

**Table 1:** Dosages and timings recommended for the prescription of D-chiro-inositol [6].

Dose	mg/Die	Time
Very Low	0–300	12+ months
Low	300–600	6 months
Medium	600–1200	3 months
High	1200+	30 days

**Table 2:** Features of patients included in the retrospective analysis at baseline and after 6 months of treatment; * *p* < 0.05; *** *p* < 0.001.

Analyses	Baseline Values	Values after 6 Months of DCI Treatment	*p*-Values
BMI (Kg/m^2^)	28.00 [26.75–29.25]	26.00 [25.00–26.25]	0.00044 ***
Glycemia (mg/dL)	105.00 [103.50–107.25]	99.00 [95.75–101.25]	0.00024 ***
Insulinemia (μIU/mL)	21.00 [18.00–24.25]	15.50 [13.00–18.25]	0.00008 ***
HOMA-IR index	4.40 [3.70–4.93]	3.00 [2.45–3.50]	0.00008 ***
FSH (mIU/mL)	7.00 [4.75–10.00]	6.95 [4.88–8.05]	0.57549
LH (mIU/L)	7.80 [5.68–10.08]	5.80 [4.90–7.43]	0.01596 *
Total T (ng/mL)	0.63 [0.33–0.80]	0.37 [0.11–0.50]	0.00010 ***
Estradiol (pg/mL)	43.50 [36.75–74.25]	62.00 [51.50–71.75]	0.03318 *
DHEAS (μg/dL)	96.50 [74.25–166.75]	79.50 [57.75–126.25]	0.00024 ***
Menstrual irregularity	4 patients	16 patients	OR = 16.00; 95% CI [3.40–75.35]

**Table 3:** Features of patients included in the prospective study at baseline and after 30 days of treatment; ** *p* < 0.01.

Analyses	Baseline Values	Values after 30 Days of DCI Treatment	*p*-Values
BMI (kg/m^2^)	20.52 [19.66–21.64]	20.56 [19.24–21.35]	0.1141
Glycemia (mg/dL)	100.00 [93.75–106.25]	98.00 [92.50–105.00]	0.96012
Insulinemia (μIU/mL)	3.13 [2.84–4.33]	2.99 [2.02–3.89]	0.57548
HOMA-IR index	0.78 [0.69–1.15]	0.75 [0.45–0.98]	0.64552
FSH (mIU/mL)	6.96 [6.11–8.87]	7.45 [6.65–10.68]	0.44726
LH (mIU/L)	5.51 [2.79–6.66]	5.97 [4.31–7.01]	0.24200
Total T (ng/mL)	0.33 [0.26–0.43]	0.58 [0.41–0.63]	0.00512 **
Estradiol (pg/mL)	37.35 [21.33–54.40]	37.15 [19.43–50.05]	0.71884
DHEAS (μg/dL)	194.20 [172.03–235.63]	211.45 [145.08–254.20]	0.50926
Asprosin (ng/mL)	1.54 [1.49–1.57]	1.96 [1.91–2.54]	0.00694 **

## Data Availability

Data can be obtained on reasonable request by contacting the corresponding author.
